# Peer Support and Crisis-Focused Psychological Interventions Designed to Mitigate Post-Traumatic Stress Injuries among Public Safety and Frontline Healthcare Personnel: A Systematic Review

**DOI:** 10.3390/ijerph17207645

**Published:** 2020-10-20

**Authors:** Gregory S. Anderson, Paula M. Di Nota, Dianne Groll, R. Nicholas Carleton

**Affiliations:** 1 Faculty of Science, Thompson Rivers University, Kamloops, BC V2C 0C8, Canada; 2 Office of Graduate Studies and Applied Research, Justice Institute of British Columbia, New Westminster, BC V3L 5T4, Canada; paula.dinota@utoronto.ca; 3 Department of Psychiatry, Queen’s University, Kingston, ON K7L 3N6, Canada; grolld@queensu.ca; 4 Department of Psychology, University of Regina, Regina, SK S4S 0A2, Canada; nick.carleton@uregina.ca

**Keywords:** post-traumatic stress injuries, mental health services, occupational health, CISD, CISM, systematic review

## Abstract

Public safety personnel (PSP) and frontline healthcare professionals (FHP) are frequently exposed to potentially psychologically traumatic events (PPTEs), and report increased rates of post-traumatic stress injuries (PTSIs). Despite widespread implementation and repeated calls for research, effectiveness evidence for organizational post-exposure PTSI mitigation services remains lacking. The current systematic review synthesized and appraised recent (2008–December 2019) empirical research from 22 electronic databases following a population–intervention–comparison–outcome framework. Eligible studies investigated the effectiveness of organizational peer support and crisis-focused psychological interventions designed to mitigate PTSIs among PSP, FHP, and other PPTE-exposed workers. The review included 14 eligible studies (*n* = 18,849 participants) that were synthesized with qualitative narrative analyses. The absence of pre–post-evaluations and the use of inconsistent outcome measures precluded quantitative meta-analysis. Thematic services included diverse programming for critical incident stress debriefing, critical incident stress management, peer support, psychological first aid, and trauma risk management. Designs included randomized control trials, retrospective cohort studies, and cross-sectional studies. Outcome measures included PPTE impacts, absenteeism, substance use, suicide rates, psychiatric symptoms, risk assessments, stigma, and global assessments of functioning. Quality assessment indicated limited strength of evidence and failures to control for pre-existing PTSIs, which would significantly bias program effectiveness evaluations for reducing PTSIs post-PPTE.

## 1. Introduction

Public safety personnel (PSP; e.g., border services officers, public safety communications officials, correctional workers, firefighters, emergency managers, operational intelligence personnel, paramedics, and police) and frontline healthcare professionals (FHP; e.g., nurses, physicians, and staff in emergency, trauma, surgical, psychiatric, geriatric, and/or intensive care units, social workers and counsellors) are regularly exposed to potentially psychologically traumatic events (PPTEs), such as threats to their own life, witnessing violence, scenes of accidents, fatalities and suicide [1,2,3,4]. PPTEs are distinct from other occupational stressors that can also impact the mental health of PSP and FHP, such as shift work, extensive public scrutiny, and workplace stigma, harassment, or bullying [5]. Despite the high rates of PPTE exposure, there are few evidence-based programs or interventions for proactively mitigating the development of post-traumatic stress injuries (PTSIs) in PSP, FHP, and other PPTE-exposed workers. The following systematic review is intended to provide various stakeholders, including worker’s compensation boards and policy makers, with an overview of the recent empirical evidence evaluating the effectiveness of post-incident services for PSP, FHP, and other PPTE-exposed workers.

PTSIs that may result from PPTE exposures include symptoms of anxiety, depression, physiological arousal, post-traumatic stress disorder (PTSD), suicidal ideation and attempts, and maladaptive coping strategies such as drug and alcohol abuse or avoidance [2,6,7,8]. In 2016, Beshai and Carleton [9] performed a comprehensive literature review on the effectiveness of peer support and crisis-focused psychological intervention programs used by tri-service agencies (i.e., firefighters, paramedics, police) to mitigate PTSIs and evaluated the available evidence of program effectiveness. The most common interventions were described as “peer support programs”, defined by Cyr et al. [10] as a supportive relationship between individuals who have experienced adverse events such as a crisis with emotional and social support, encouragement, and hope. Other common interventions included “crisis-focused psychological intervention programs”, the most common being critical incident stress debriefing (CISD), which is generally implemented 24–72 h following exposure to a PPTE identified as critical. CISD is typically intended to provide opportunities for assistance and support in the context of work-related stressors [9,11]. The authors concluded there was “limited availability of research evidence and the important limitations in the existing research make conclusive decisions regarding the use of such programs impossible” [9] (p. 8). Likewise, results of a meta-analysis assessing the impact of police-specific stress management interventions designed to improve psychological, physiological, and behavioral outcomes appeared to evidence that the “interventions had no significant effect on … outcomes” [12] (p. 6).

A high prevalence of violent workplace exposures has also been described among FHP, with between 9 and 56% of respondents indicating exposure to some form of workplace violence in the previous 12 months, including physical violence and verbal aggression [13,14,15,16,17,18]. Accordingly, the rates of PTSD among various FHP occupational groups reportedly range between 8 and 29% [18,19,20,21]. What remains unexplored in the literature are studies investigating the effectiveness of services designed to mitigate risk of PTSIs following a PPTE and tailored to the unique occupational needs of FHP.

The current study is a systematic review of the recent literature (2008–2019) investigating the effectiveness of organizational peer support and crisis-focused psychological interventions intended to mitigate PTSIs among PSP, FHP and other relevant groups at risk of occupational PPTE exposure. The various programs or interventions identified in eligible studies are qualitatively summarized, including intended study goals, employed approaches, durations, and outcome measures, and principal findings. The quality and strength of research evidence is also assessed. The current synthesis of services and programs delivered after PPTE exposure can inform the effective development, implementation, evaluation, and evidence-based provision of intervention strategies that maximally mitigate PTSIs among PPTE-exposed workers.

## 2. Materials and Methods

### 2.1. Protocol and Registration

The current study was pre-registered with PROSPERO (CRD42019133534) [22]. The systematic literature review procedures followed PRISMA guidelines [23].

### 2.2. Eligibility Criteria

Eligibility was restricted to English- or French-language studies exploring the use of peer support and crisis-focused psychological interventions used to mitigate sequalae from PPTE exposures among adult (aged 18 and older) PSP and FHP. Eligible PSP occupations were border services officers, correctional workers, communications officials (e.g., dispatch operators, 911 operators), firefighters, paramedical professionals, and police. FHP occupations included nurses and personnel working in emergency rooms, trauma centers, and surgical teams, social workers and counsellors. Other occupations recognized to experience a high risk of traumatic exposures were also considered, such as emergency management response teams and rail transit operators. Eligible studies could be of any length of follow up, from any geographic location, but the search was restricted to studies published in 2008 onwards. Exclusion criteria included study protocols, qualitative studies, case studies, investigations that tested the acceptability of a service among its participants, and investigations on the effectiveness of a service on job-related satisfaction without evaluating outcomes of interest (i.e., sickness absence, mental health symptoms, suicide rates).

### 2.3. Information Sources and Literature Search

There were 22 electronic databases searched from 2008 to 9 December 2019, including PsycINFO, PubMed, JSTOR, Web of Science and Wiley, Sage, Taylor & Francis, Cambridge and Oxford journal online. The electronic yield of records was supplemented with hand searches of the reference lists of included studies, with selected articles searched in Google Scholar. Key terms used for database searches were derived from a population–intervention–comparison–outcome (PICO) framework (see Table 1).

### 2.4. Study Selection

After the search was completed, all citations were imported into Covidence—a web-based systematic review manager [24]. Initial screening at the title/abstract stage was verified by having multiple reviewers screen the same 200 papers, with 99% agreement. There were two reviewers who then screened full papers to determine acceptability for inclusion in the systematic review. All discrepancies were resolved by consensus between the two reviewers. Data were extracted from the published full-text reports of each included article independently by two reviewers. The data extraction was facilitated by customized tables developed in Covidence directly.

### 2.5. Data Items

A PICO framework was used to define variables for which data were sought. Population variables included the sample size, age, sex, and years of employment in their profession. Intervention variables included the type and duration of program (e.g., critical incident stress management or debriefing, peer support training, suicide prevention, and timing and frequency of individual or group sessions). Comparison variables included the type and nature of the comparator group (e.g., waitlist controls or within-subject analysis of pre- and post-training measures). Outcome variables included rates of absenteeism, scores on self-report instruments for stress, burnout, resilience, and symptom-based measures of mood disorders, anxiety disorders, PTSD, and other PTSIs, such as the Depression Anxiety Stress Scale-21 (DASS-21) [25]. Physiological markers of stress were also included as outcome variables where available (e.g., heart rate, blood pressure, salivary and plasma cortisol), as were number of missed workdays.

### 2.6. Quality Assessment in Individual Studies

The 9-item Newcastle–Ottawa quality assessment scale for cohort studies was applied to assess study quality and the strength of research evidence in individual studies [26]. Each study was evaluated on three domains (i.e., selection, comparability, outcome) and received a rating for a low or high risk of bias for each of nine items (i.e., a low risk of bias counts as one point) for a total possible score of nine. Items where a low or high risk of bias could not be determined received an ‘unclear’ rating and were counted the same as a high risk of bias. While there is no established standard for interpreting total quality assessment scores, the current study will classify a total score of 9 as ‘high quality’, scores of 7 or 8 as ‘moderate to high quality’, scores of 5 or 6 as ‘moderate to low quality’, and scores below 5 as ‘low quality’.

## 3. Results

Data were qualitatively synthesized using descriptive tables to summarize the design, characteristics, and outcomes of each study (Table 2). Individual studies were grouped using thematic analysis into broad categories to facilitate meaningful discussion points. The capacity for a quantitative meta-analysis was precluded by the diverse nature of studies considered and outcomes reported.

### 3.1. Study Selection

**Figure 1:**
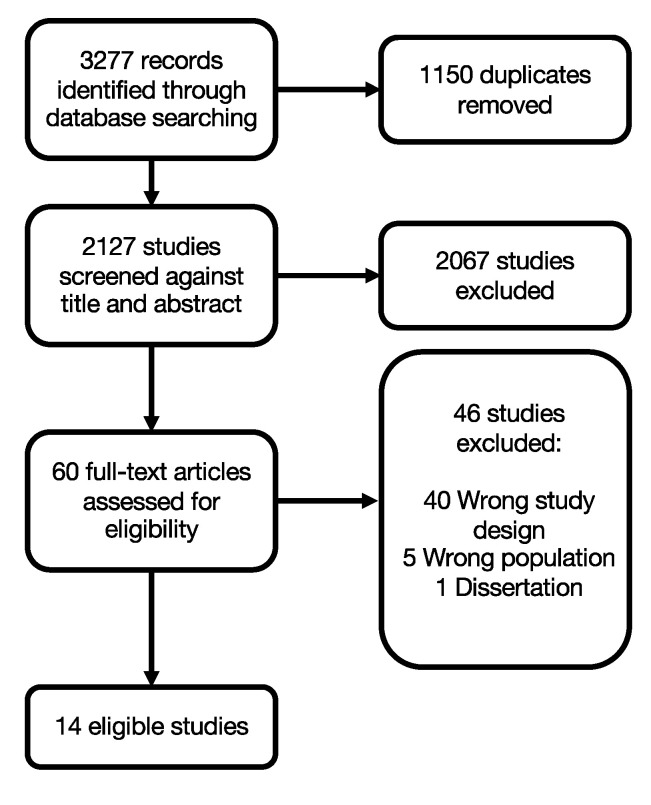
PRISMA flow diagram.

There were 3277 records identified from a systematic literature review. There were 1150 duplicates removed, leaving 2127 studies for screening. There were 2067 records removed by title/abstract screening, leaving 69 studies for full-text review. There were 46 studies removed at the full-text stage (i.e., 40 had the wrong study design, 5 had the wrong population (military), and 1 was a dissertation). The systematic review process resulted in 14 eligible studies (Figure 1). The inconsistency in pre–post-evaluations and for measured and reported outcomes across studies made a quantitative meta-analysis on service effectiveness impossible. Therefore, studies are thematically categorized and described below, followed by quality assessment of the strength of evidence across studies.

### 3.2. Description of Studies

The PSP professions represented in the available studies were fire and rescue (including officers, volunteer firefighters, and duty managers) (*n* = 5) and police (including sworn and former officers, union representatives, and civilian employees) (*n* = 5); no eligible studies pertaining to other groups of PSP were identified. FHP included nursing students completing a practical unit (*n* = 1), personnel in pediatric liver transplant centers (*n* = 1), and healthcare workers in large general hospitals (*n* = 1). The only other relevant occupation group represented in eligible studies included PPTE-exposed public transport operators (*n* = 1). In total, 18,849 individuals were represented across studies. There were eight studies that explicitly evaluated PPTE exposure or offered their respective service following an occupational PPTE. The eligible study criteria for the current review (i.e., organizational services offered to buffer the negative psychological effects of experienced or future PPTEs) allowed for PPTE exposure to be inferred for the remaining six studies based on participant occupations [1,2,3,4].

Thematic groups identified within the literature included CISD (*n* = 5: included 2 studies with undefined organizationally-offered or -facilitated debriefing) and critical incident stress management (CISM, *n* = 1), as well as several peer support programs (*n* = 8) including types of psychological or mental health first aid and trauma risk management. Study designs included randomized control trials (RCTs) and cluster RCTs (*n* = 4), retrospective cohort studies (*n* = 4), a prospective cohort study (*n* = 1), and cross-sectional studies (*n* = 5). Control interventions included waitlist controls (*n* = 2), psychoeducation only and no peer support training (*n* = 1), or group versus video versus control versions of the intervention (*n* = 1). Comparisons included regular training or service as usual, or alternative physical health or general wellness-focused interventions (*n* = 3). The duration of services or training sessions were commonly not reported (*n* = 7), but reported services were administered for 60 min beginning within an hour of the PPTE concluding [40] or for approximately 90 min within three days of the PPTE concluding [31]. The training program durations were 90 min [35], 4 h [33,37], 13 h [32], or two full-day sessions [38]. Study duration for RCTs and retrospective cohort studies ranged from one month [31] to 22 years [38].

### 3.3. Effectiveness of Critical Incident Stress Debriefing (CISD)

There were five studies that reported results of CISD or related debriefing (two studies with undefined organizationally-offered debriefing), of which three involved firefighters, one involved police, and one involved allied health professionals (see Table 2). There were four cross-sectional or retrospective cohort designs with measurement at only one point in time. Tuckey and Scott [31] used an RCT to compare results of the Mitchell model group CISD with groups who received stress management education or screening only (control group). The results indicated no statistically significant differences in PTSI symptoms between groups at pre- or post-intervention, and a reduction in alcohol consumption one-month post-intervention for the active groups relative to the control group was not sustained at follow up. There were three studies that reported no statistically significant differences in mental health outcomes between those who did and did not have access to debriefing [28,29,30]. There was one study [27] that reported participants (*n* = 57) who received organizationally-offered or -facilitated 90 min debriefing (having to attend a group session, individual meeting with a psychologist, or both) reported higher perceived event-related stress and PTSD scores than non-debriefed participants at a 5 year follow up.

### 3.4. Effectiveness of Critical Incident Stress Management (CISM)

There was one study [40] that reported on the introduction of 60 min of CISM offered within 90 min of a PPTE within a healthcare setting; however, the study found that there was little consistency with respect to the application of CISM. The study by Müeller-Leonhardt and colleagues [40] had a low response rate (17.6%: *n* = 88), only 25% of potential post-PPTE participants were offered post-incident CISM, and no mental health measures were collected. Therefore, the study offers no data to assess CISM program effectiveness.

### 3.5. Effectiveness of Peer Support Programs

There were eight studies that offered peer support programs for various outcomes (mental health and suicide prevention) and within various populations including police (*n* = 4), healthcare (*n* = 1), fire services (*n* = 2), and transportation (*n* = 1). There were three RCTs [32,35,37], each using different outcome measures (mental health, increased use of peer support services, sick leave), but all reporting favorable results. In a prospective cohort study, Carleton et al. [33] reported short-lived, small, but statistically significant improvements in stigma following the Road to Mental Readiness training program, but no statistically significant improvements in mental health. The two retrospective cohort studies [34,36] examined sick days as an outcome measure, while one study [38] examined suicide rates. Again, all studies reported favorable results, with varying quality of research and strength of evidence. Finally, Watson and Andrews [39] used a cross-sectional study design and found evidence for improved mental health scores with fewer PTSI symptoms as measured by standardized tools and fewer barriers to care for police officers who worked within a force who received trauma risk management training.

### 3.6. Quality Assessment

**Figure 2:**
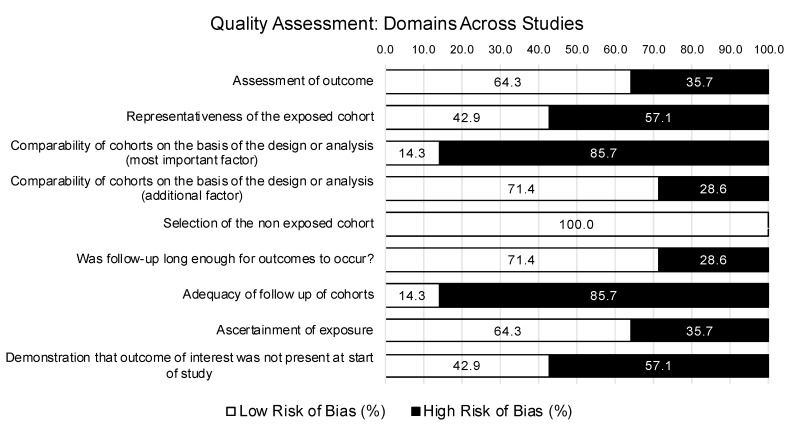
Quality assessment for strength of research evidence using the Newcastle–Ottawa quality assessment scale for cohort studies—full sample summary (*n* = 14 studies).

A summary of study quality ratings is illustrated in Figure 2. According to the interpretation standards established for the current review, none of the 14 studies were classified as high quality. All of the 14 studies received at least one high risk or unclear rating on the strength of evidence criteria. One study was rated moderate to high quality [35], nine studies were of moderate to low quality [27,31,32,33,34,36,37,38,39], and four studies were of low quality [28,29,30,40].

#### 3.6.1. Outcome

There were 9 of 14 studies rated at a low risk of bias regarding the assessment of study outcomes based on the use of secure organizational records (e.g., rates of sickness absence and suicide) or empirically-validated mental disorder screening tools. There were five studies rated at a high risk of bias for using revised versions of previously validated measures [31,32] or unvalidated self-report measures [29,30,40], which could be prone to individual reporting biases (e.g., memory errors, desire to respond in a favorable way that minimized stigmatized attitudes or behaviors).

Except for four cross-sectional studies [28,29,39,40], all remaining studies provided sufficient time following participation in a PTSI mitigation service or program before collecting outcome measures, resulting in low risk of bias ratings based on time.

There were 12 out of 14 studies that were rated as high risk (*n* = 3) or unclear (*n* = 9) regarding adequacy of follow up due to study design (e.g., cross-sectional or retrospective cohort studies), precluding measurements at more than a single point in time and precluding any valid assessment of service effectiveness. The remaining RCTs [31,37] or prospective cohort designs [33] that did conduct follow-up measures received high risk of bias ratings for failing to provide an analysis of baseline measures and/or demographic variables between participants lost at follow up and those who completed follow-up measures; however, Carleton and colleagues [33], and Tuckey and Scott [31] did apply appropriate statistical analyses (i.e., multilevel hierarchical modelling) to account for post-intervention attrition.

#### 3.6.2. Selection

Half of the studies included in the current review did not demonstrate that their sample was representative of the larger population of workers with respect to demographic variables such as sex, average age, or years of service, limiting generalizability of their results. All studies were rated at a low risk of bias regarding selection of the non-exposed cohort, which was either randomly selected from the same population in the case of RCTs [31,32,35,37], or compared to a sample from the same larger population that did not offer the service in question [27,34,36,38], or not applicable for single-sample cross-sectional and prospective cohort study designs [28,29,30,33,39,40]. There were four studies rated a high risk of bias due to participants self-reporting prior participation in, or exposure to, a given intervention [27,28,30] or due to a substantial proportion of the sample (41%) being unaware of the availability of the service prior to taking part in the study [40]. There was one study that received an unclear rating based on the study outcome, which measured participants’ preference for, and not exposure to, various formal (e.g., CISD) and informal debriefing procedures [29].

#### 3.6.3. Comparability

Most studies (12 of 14) were deemed at a high risk of bias for failing to control for, or account for, the most important factor in the study design or analysis—the presence of a PTSI or diagnosable mental disorder at the time of the study—which would substantially bias the study outcome (i.e., evaluating the effectiveness of a PTSI mitigation service). Similarly, 8 out of 14 studies received a high-risk rating for failing to demonstrate that participants were apparently healthy at the start of the study and not already suffering from PTSIs or PTSD. Most studies (10 of 14) were at a low risk of bias for controlling for an additional factor in their study design or analysis, such as participant sex, age, and/or years of service, which have been statistically significantly associated with PSP mental health outcomes.

## 4. Discussion

PSP and FHP are regularly exposed to PPTEs, such as threats, violence, accidents, fatalities, and suicide, as well as occupational stressors (e.g., shift work, public scrutiny, harassment or bullying) [1,2,3,5,6]. PTSIs resulting from PPTEs include symptoms of mood and anxiety disorders, as well as other mental disorders (e.g., PTSD), suicidal behaviors (i.e., ideation, planning, attempts), and maladaptive coping strategies (e.g., drug abuse, alcohol abuse, avoidance) [2,6,7,8]. The impact of PTSIs may include a reduction in the quality of occupational performance, increased absenteeism, sleep difficulties, a negative impact on relationships with others, burnout, other physical or psychological illnesses, disability, and early mortality [5,41,42,43]. The economic burden of PTSIs within PSP and FHP in Canada remains unknown, but productivity losses that result from mental disorders experienced in the Canadian workforce are estimated to be anywhere between $16.6 billion [44] and $51 billion [45,46,47] annually. Especially in light of the global novel coronavirus pandemic, identifying effective programs and services that can change the occupational health trajectories of PSP and FHP following PPTEs, and mitigate PTSIs, is imminently required.

Several discrete programs have been developed as part of efforts to mitigate the impact of PPTEs in both PSP and FHP. Most of the programs involve very diverse peer support and crisis-focused psychological interventions. As evidenced in the current review, the programs and any associated evaluations have varied greatly in study design, target audience, duration of training, timing of intervention, outcomes measured, and timing of follow up. Comparing the effectiveness of programs with such diverse elements is extremely difficult, and quality assessments of the impact such programs may have on mental health and absenteeism of participants post-PPTE are rarely available. Nevertheless, the available programs can be broadly generalized into “peer support” and “crisis-focused” psychological interventions [9]. The most common, but diverse, interventions are described as “peer support programs”, which rely on trained peers to create a supportive relationship with individuals who have experienced adverse events with emotional and social support, encouragement, and hope [10]. Crisis-focused psychological intervention programs typically refer to a wide variety of CISD or CISM derivations, offering problematically diverse direct support programming post-PPTE exposure, often using the same name to describe very different programming. The assessed interventions may be conducted with a trained mental health professional or service provider and offer a time-limited (typically 24–72 h) intervention post-PPTE [9].

The current review identified 14 studies measuring the effectiveness of peer support programs and crisis-focused psychological interventions among PSP and FHP following exposure to a PPTE with the hopes of mitigating PTSIs, and ultimately PTSD. As the associated extent of literature is still early in development, the ability to draw conclusions about a particular service or intervention that is most effective for mitigating PPTE sequela exceeds the available data; nevertheless, a few themes are apparent across the available studies. First, some administrations of the diverse programs often synonymously referred to as CISD may be beneficial, but the evidence remains insufficient; relatedly, some forms of organizationally-offered or -facilitated CISD may be problematic, but the evidence remains grossly insufficient. Second, given the heterogeneity in results and effectiveness across PSP and FHP, a “one-size-fits-all” approach may not be ideal. Finally, while there was a diverse group of programs developing peer support, there is very preliminary evidence supporting peer support as associated with at least short-term favorable results. To facilitate iterative independent evaluation by researchers, established and transparent programs should be consistently applied, have defined structures (i.e., evidence-informed content and prescribed durations and evaluation intervals), and support fidelity and fidelity assessments. The results of such rigorous investigations into service effectiveness would in turn support evidence-based practices, profession-specific tailoring, and progressive improvements to PTSI mitigation strategies for at-risk occupational groups.

### 4.1. Significance of Results

There is substantial evidence for a variety of psychotherapies established for the treatment of conditions such as PTSD that may result from work-related PPTEs, including PPTE-focused cognitive behavioral therapy, cognitive restructuring and cognitive processing therapy, and prolonged exposure, eye-movement and desensitization reprocessing [48]. Comparatively, there is a dearth of literature examining the effectiveness of proactive strategies for mitigating PTSIs following PPTE exposure [49]. Given that PSP and FHP appear at greater risk for PPTE exposures, the identification of effective post-exposure strategies for mitigating PPTE-related disorders would be a substantial achievement. Increasingly, studies have explored the unique mental health needs of PSP using a PPTE-informed lens. There is still a dearth of studies specifically focusing on PSP from a treatment and programming perspective.

Beshai and Carleton [9] characterized the timing of peer support and crisis-focused psychological intervention programs as before, during, or after a crisis, with some programs (e.g., peer support, CISM) being offered at all three times. The format of the interventions varied between group and individual programs, with most interventions offering both. Providers varied between mental health professionals, peer support personnel, community members, social workers, and PSP team leaders. Paralleling the current results, summarizing the results of programs and interventions reviewed, the authors concluded that there was “limited availability of research evidence and the important limitations in the available research make conclusive decisions regarding the use of such programs impossible” [9] (p. 8).

The current results are similar to results from work performed with general population samples. Forneris and colleagues [50] and Forman-Hoffman and colleagues [51] found limited evidence supporting whether timing, intensity, and dosage impacted the effectiveness of post-PPTE programs designed to mitigate PTSIs, and whether outcomes from early interventions were impacted by demographic characteristics, psychiatric comorbidities, and personal risk factors. Their review evidenced that studies were limited by small study sizes, high attrition rates, and methodological shortcomings (e.g., absent randomization), problematic statistical methods, and a high risk of bias [50,51,52]. The current review also found inconsistent reporting of methodological approaches, outcome measures, and potential confounds to program effectiveness, including pre-existing PTSIs or mental health conditions, symptom duration and/or severity, and concurrent treatment.

The limited evidence available is favorable towards peer support programs, with small, but potentially important, short-term results. Studies have inconsistently demonstrated increasing mental health knowledge as being associated with less stigmatic attitudes towards self and others [5,30], and more confidence for recognizing when a peer may need help with basic skills such as starting a conversation out of concern for others or supporting help-seeking behavior [5,35], with peer support research deserving further exploration [5]. There are studies indicating that mental health training is associated with increased participants’ knowledge regarding mental health, decreases in their negative attitudes, and increases in supportive behaviors toward individuals with mental health problems [5,53]; however, due to a lack of consistent outcome measures, there is still no way to understand whether any services significantly change the mental health trajectory of PSP and FHP following PPTE exposure.

### 4.2. Strengths and Limitations

The current review provides a recent update on past studies exploring the use of services designed to mitigate psychological sequelae among PSP and FHP, focusing on the last 10 years of research. The broad search strategy and inclusive eligibility criteria facilitated the identification of studies encapsulating a broad range of service types and classes of PSP and FHP. There are also several key limitations that can inform directions for future research. As a systematic review, many of the strengths and limitations of the present study are intimately tied to the nature of the available component studies. Excluding studies published prior to 2008 reduced the yield and our capacity for quantitative meta-analysis. The broad inclusion criteria—while helpfully increasing the component studies available for the current review—substantially increased heterogeneity. Consequently, for any particular group of PSP, FHP, or other PPTE-exposed workers, there were at most a few studies.

### 4.3. Future Research

Additional studies are needed for understanding the potential impact of peer support and crisis-focused psychological intervention programs for PSP, FHP, and other workers frequently exposed to PPTEs. Future studies need to (1) use standardized outcome measures, (2) control for persons with a pre-existing PTSI among participants receiving interventions intended to mitigate PTSI development, and (3) use methods sensitive to changes over time. Unfortunately, 12 of 14 studies reviewed were cross-sectional or retrospective cohort studies, precluding discussions of causation. Future studies could also directly compare the effectiveness of different programs for different groups of workers using standardized outcome measures. Additionally, large studies with longer follow-up periods are needed to determine the longevity of benefits over time. For example, Carleton and colleagues [33] reported a small, but temporary, decrease in stigma following implementation of one version of the four-hour Road to Mental Readiness (R2MR) course; however, the use of skills from the course declined at 6 and 12 month follow ups. The current recommendations align with previous recommendations, such as those of the 2008 Australian government in “An organizational approach to preventing psychological injury”, which emphasized the need to monitor and review the implementation and effectiveness of interventions using agreed upon performance indicators and targets to ensure continuous improvement [54].

Future researchers should also pay close attention to the symptomology developed by each population of interest following occupational exposure to PPTEs, including the type, duration, and severity of PTSI symptoms, as well as any concurrent treatment. Together with comparable outcome measures, more comprehensive reporting of PTSI symptoms and PPTE exposures will further elucidate program effectiveness with greater scientific quality and rigor.

## 5. Conclusions

There is inconsistent evidence for the effectiveness of several organizational services developed and deployed to mitigate the psychological impact of PPTEs among PSP, FHP, and other workers frequently exposed to PPTEs. Despite the lack of evidence, several organizations have implemented the crisis-focused psychological interventions and peer support services presently reviewed [5,9,46]. The broad variety of occupational populations sampled, intervention approaches implemented, and outcomes evaluated in the current review preclude denoting any service as superior to any other for mitigating PTSIs. With numerous forms of every program, including CISD, each with different fidelity challenges with respect to application, and fundamental problems with study design and consistency of outcome measures, recent evidence of the effectiveness of post-PPTE crisis-focused interventions for PSP and FHP is sorely lacking and inconclusive. Similarly, with the wide breadth of peer support programs observed and large variability in outcomes measures (many of which are unrelated to PTSI mitigation), there is low to moderate evidence to support their use with PSP and FHP.

Despite the important contemporary efforts, there currently remains a substantial gap in research and peer-reviewed literature on the effectiveness of organizational programs, interventions, and services, as well as educational programs intended to reduce PTSIs following PPTE exposures among PSP and FHP. As policy makers mobilize legislation for mental health services across sectors in response to the global coronavirus pandemic, formal evaluation of the effectiveness of the proffered services is needed through careful and rigorous independent research inquiry, especially for evaluating the suitability and effectiveness of services tailored to PSP and FHP.

## Figures and Tables

**Table 1:** Key terms used for database searches.

Domain	Target	Search Terms
Population	Public safety personnel	Public safety personnel First responder Emergency personnel Police Firefighter or fire fighter Paramedic or ambulance personnel, or emergency medical service or emergency medical technician Dispatcher Correctional officer Nurse
Intervention (Services)	Post-exposure services	Peer support Spousal or family support Psychological first aid Mental health first aid Pastoral crisis intervention Critical incident stress management or CISM Critical incident stress debriefing or CISD Crisis management debriefing Debriefing Defusing Family CISM Post-traumatic stress management Couples overcoming PTSD everyday (COPE) OSI Canada family program
Condition	Post-traumatic stress injuries	Mental health Psychological distress Psychological injury Critical incident Occupational stress Posttraumatic stress injury or post-traumatic stress injury or PTSI Posttraumatic stress disorder or post-traumatic stress disorder or PTSD

**Table 2:** Eligible studies investigating organizational services for public safety, frontline healthcare, and public transport professionals following potentially psychologically traumatic exposures.

Study	Sample Size	Population (Country)	Design	Intervention Description	Intervention Duration	Study Duration	Outcomes	Results
**Informal Organizationally-Offered or Organizationally-Facilitated Post-Incident Debriefing and Formal Critical Incident Stress Debriefing (CISD)**								
Addis and Stephens, 2008 [27]	57	Sworn, civilian, and former police workers (New Zealand)	Retrospective cohort study	Received organizationally-offered or -facilitated debriefing (attended group session, individual meeting with psychologist, or both) vs. no debriefing	Not provided	5 years after murder of on-duty officer and manhunt	Perceived stress of event, IES-R, GHQ, TSS, PSS	5 years following a PPTE, only 21% (12 of 57) of respondents received organizationally-offered or -facilitated debriefing, and reported higher perceived stress of the event and PTSD scores than non-debriefed participants
Duncan et al., 2018 [28]	120	Allied health professionals (physicians, nurses, mental health professionals) in pediatric liver transplant centers (USA)	Cross-sectional study	Formal organizational debriefing procedures vs. no debriefing	Not provided	N/A	MBI-EE, Bereavement Experiences Scale, Guilt/Blame/Anger subscale (not reported)	Significantly less EE among respondents who indicated they had formal debriefing procedures at their organizations compared to those without formal debriefing following the death of a patient. No significant differences in outcomes between those who did and did not have access to other types of support (i.e., bereavement or coping training or guidelines, support staff, informal support). Results not reported for individuals who have (*n* = 83) and have not (*n* = 37) received support (formal or informal); authors contacted for data
Jeannette and Scoboria, 2008 [29]	142	Firefighters (Canada)	Cross-sectional study	CISD vs. individual debriefing vs. informal discussion vs. no intervention	Not provided	N/A	Preference rating for each type of intervention following five scenarios increasing in severity	Firefighters expressed interest in working within their peer group for all events, and an increasing interest in formal intervention as event severity increased. Individual debriefing was preferred to CISD in scenarios of low to moderate intensity, and all interventions were of high interest for high intensity scenarios. Means and SDs for preference ratings for each scenario type not provided, requested from authors
Sattler et al., 2014 [30]	286	Firefighters (USA)	Cross-sectional study	CISD	Not provided	N/A	Number of critical incident exposures in their career, attendance and experience with CISD, burnout, post-traumatic stress symptoms (past 30 days), post-traumatic growth inventory, problem- and emotion-focused coping and disengagement	94% of respondents indicated exposure to a critical incident during their career, 52% participated in CISD, and 64% of these participants reported stress reduction 2 weeks after attending. Having a positive attitude toward CISD was positively associated with post-traumatic growth but not related to post-traumatic symptoms. Participants indicated they receive support from co-workers and family, and reported minimal burnout. Purely descriptive study, no comparison between groups or over time
**Psychological and/or Mental Health First Aid and/or Peer Support Programs**								
Tuckey and Scott, 2014 [31]	67	Volunteer firefighters (Australia)	RCT	Mitchell model group CISD vs. stress management education vs. screening only (no treatment control)	90 min CISD and education sessions within three days of the PPTE (motor vehicle accident, failed resuscitation)	1 month follow up	IES-R, K-10, quality of life enjoyment and satisfaction questionnaire, past week alcohol consumption	Mean levels of post-traumatic stress (IES-R) and psychological distress (K-10) were generally low and did not differ between groups pre- or post-intervention. Controlling for pre-intervention scores, CISD was associated with significantly less alcohol consumption one-month post-intervention relative to the screening only condition, but not the education group, and higher post-intervention quality of life compared to the education but not screening only group
Burns et al., 2017 [32]	181	First-year nursing students completing a practical unit (Australia)	RCT	MHFA vs. waitlist controls	2 × 6.5 h courses	2 months	Mental health knowledge, confidence, first aid intentions, stigmatizing attitudes towards self and others, SDS	Significant improvement on all outcome measures in the MHFA intervention group only. Only means for outcome measures are reported; author contacted for SD values
Carleton et al., 2018 [33]	133	Police officers (Canada)	Prospective cohort study	Psychoeducational resilience promotion, stress management, coping skill building (R2MR)	4 h course	Immediately post-training, and at 6 and 12 months	BRS, DASS subscales, PCL, AUDIT	No change in mental health or resilience outcomes post-training, or at 6 or 12 month follow up, but small significant reduction in stigma post-training
Clarner et al., 2017 [34]	259	Public transport operators (Germany)	Retrospective cohort study	PFA peer support by colleagues vs. PFA peer support by supervisors vs. no intervention	Not provided	180 days following the PPTE (accident, attack, collision, suicide)	Sickness absence in days after the PPTE	Descriptive and regression analyses explore numerous situational factors that contribute to sickness absence in each group, but data provided are not useable in the present meta-analysis
Gulliver et al., 2016 [35]	172	Firefighters and officers (USA)	RCT	Reach Out group intervention vs. Reach Out video intervention vs. health video control intervention	90 min	3 months	Attempts to intervene with a colleague in distress, number of successful interventions in the past 3 months, intervention effectiveness, treatment adherence	Participants in the Reach Out video condition reported a significant increase in successful interventions and intervention effectiveness from pretest to the 3 month follow up compared with the control group. Individual group means and SDs requested from author
Hunt et al., 2013 [36]	210	Police officers (England)	Retrospective cohort study	TRiM peer support and risk assessment intervention	Not provided	2 month period following the PPTE (multiple fatality incident)	TRiM risk assessment score, sickness absence	Significant reduction in TRiM scores for individuals who received additional treatment from the agency clinical psychologist (36 of 210) compared to the untreated group. Means and SDs for sickness absence by various treatment groups not provided, authors contacted
Milligan-Saville et al., 2017 [37]	44 managers of 1966 employees	Fire and rescue duty managers (Australia)	Cluster RCT	RESPECT manager training program vs. WLC	4 h face-to-face group session	12 months (6 months preceding and following training)	Change in rate of work-related and standard sickness absence of reporting personnel 6 months before and after the program	Work-related sick leave decreased among employees for managers in the training group, and increased in the control group. Standard sick leave rates increased among both groups, perhaps due to follow-up period being in the winter months
Mishara and Martin, 2012 [38]	14,309	Police officers, supervisors and union representatives (Canada)	Retrospective cohort study	Together for Life suicide prevention and peer support program administered to Montreal police service (*n* = 4178) vs. no training among all other Quebec provincial police (*n* = 10,131)	2 × half-day suicide awareness and support session + full-day session for supervisors and union reps led by psychologist in 2000–2001 and 2006	22 years	Police suicides in the ten years preceding (1986–1996) and 12 years following (1997–2008) training	The Montreal police suicide rate decreased significantly by 78.9%to 6.42/100,000 per annum, while the other Quebec police had an 11.4% non-significant increase in suicides to 29.0/100,000; significant post-program difference between Montreal and other provincial police suicide rates
Watson and Andrews, 2018 [39]	859	Police employees (UK)	Cross-sectional study	TRiM vs. no TRiM	Not indicated	N/A	PCL-C, Stigma and Barriers to Care Questionnaire, MSS self and public stigma subscales	Participants in forces that offer TRiM reported significantly less public stigma and fewer post-traumatic symptoms and barriers to care compared to participants in forces that do not offer TRiM or any standardized PPTE support or process
**Critical Incident Stress Management (CISM)**								
Müeller-Leonhardt et al., 2014 [40]	88	Healthcare workers (Germany)	Cross-sectional study	CISM vs. untrained staff	60 min within an hour of the PTE	N/A	VAS% for contributing factors to critical incident recovery, sources of support for coping with critical incident symptoms	Non-CISM personnel rated family and colleagues as primary sources of support and spontaneous recovery as the greatest contributing factor, while CISM peers endorsed the program and peers. 41.3% of the sample had only learned about CISM via the current study’s survey. Only 36 participants responded to a question regarding CISM following a PPTE, and 75% of these stated they had not been offered post-incident CISM

Note: RCT: randomized control trial; WLC: waitlist control; CISD: critical incident stress debriefing; CISM: critical incident stress management; MHFA: mental health first aid; PFA: psychological first aid; TRiM: trauma risk management; N/A: not applicable; IES-R: Impact of Events Scale Revised; GHQ: General Health Questionnaire; K-10: Kessler-10; MBI-EE: Maslach Burnout Inventory Emotional Exhaustion Subscale; MSS: Military Stigma Scale; PCL-C: Post-traumatic Stress Disorder Checklist; PPTE: potentially psychologically traumatic event; PSS: Police Stress Survey; SDS: Social Distance Scale; TSS: Traumatic Stress Schedule; SD: standard deviation; VAS: visual analog scale.
